# Cancer outcomes and cardiopulmonary toxicities for Black patients with breast cancer treated with proton therapy

**DOI:** 10.1093/jncics/pkae129

**Published:** 2024-12-28

**Authors:** Gurbani Singh, Sravya Koduri, Manaahil Rao, Meira Kidorf, Sarah Ruff, Akshar Patel, Søren M Bentzen, Elizabeth Nichols, Sarah McAvoy, Melissa A L Vyfhuis

**Affiliations:** Department of Radiation Oncology, University of Maryland School of Medicine, Baltimore, MD 21201, United States; Department of Radiation Oncology, NYU Langone Health, New York, NY 10016, United States; Department of Radiation Oncology, University of Maryland School of Medicine, Baltimore, MD 21201, United States; Department of Radiation Oncology, University of Maryland School of Medicine, Baltimore, MD 21201, United States; Department of Radiation Oncology, University of Maryland School of Medicine, Baltimore, MD 21201, United States; Department of Radiation Oncology, University of Maryland School of Medicine, Baltimore, MD 21201, United States; Department of Radiation Oncology, Chesapeake Oncology-Hematology Associates, Glen Burnie, MD 21061, United States; Department of Epidemiology and Public Health, Biostatistics and Bioinformatics Division, University of Maryland School of Medicine, Baltimore, MD 21201, United States; Department of Radiation Oncology, University of Maryland School of Medicine, Baltimore, MD 21201, United States; Department of Radiation Oncology, University of Maryland School of Medicine, Baltimore, MD 21201, United States; Department of Radiation Oncology, University of Maryland School of Medicine, Baltimore, MD 21201, United States

## Abstract

**Background:**

Black women have a 40% higher breast cancer mortality rate than White women and are at a higher risk of acquiring cardiovascular disease. Proton therapy can be used to mitigate cardiac radiation exposure; however, proton therapy remains a scarce resource in the United States. We report on the cardiovascular profiles of patients undergoing proton therapy to determine the potential benefit of the therapy for Black women compared with patients of other races.

**Methods:**

We retrospectively analyzed 599 patients with breast cancer who received proton therapy from June 2016 to December 2021 at the Maryland Proton Treatment Center. A variety of sociodemographic, disease, and treatment variables were analyzed using descriptive statistics.

**Results:**

With a median follow-up of 26 months (range = 0.47-90 months), Black patients made up 31.6% of the population and presented with higher rates of hypertension (*P* < .001), cardiopulmonary conditions (*P* < .001), and a higher median body mass index (*P* = .015) compared with the other cohort, a trend that persisted at the time of post–proton therapy follow-up. Black women had higher rates of triple-negative disease (*P* < .001), with subsequent greater receipt of neoadjuvant chemotherapy (*P* = .039). Pulmonary events were 2.6 times more likely to occur in Black patients than in the non-Black cohort after proton therapy (odds ratio = 2.60, 95% CI = 1.39 to 4.88; *P* = .003).

**Conclusions:**

Black women presenting for proton therapy had higher baseline risks of cardiovascular co-morbidities combined with more aggressive breast cancer biology and a subsequent 2.6-fold increased risk of pulmonary events after proton therapy. Our findings support the use of advanced radiation techniques as a means of sparing important organs at risk, especially in historically marginalized populations.

## Introduction

Racial inequities in female breast cancer mortality rates have persisted since the dissemination of early detection and treatment advances in the 1980s.^1^ Despite lower incidence rates, Black women have a 40% higher breast cancer mortality rate than White women, and among women younger than 45 years of age, the rate more than doubles.^2^^,^^3^ Contributing factors to such high mortality rates in Black women include a higher proportion of distant-stage disease at diagnosis and unfavorable and more aggressive tumor characteristics, such as triple-negative disease and inflammatory carcinoma, than women of other races.^3^ Although genetic factors may explain some of the inequities present in Black women with breast cancer, several studies have reported on additional aspects, such as socioeconomic status and inequitable access to high-quality screening and treatment facilities, that can further exacerbate negative cancer outcomes.^3-6^

Furthermore, Black women tend to have a higher risk of acquiring cardiovascular disease (CVD) than women of other races, developing CVD at a younger age, with an associated higher mortality rate.^7-9^ Stark differences in mortality also exist between racial cohorts in terms of hypertension, stroke, congestive heart failure, and coronary artery disease, where Black women with coronary artery disease have a 69% increased risk of relative mortality compared with White women. Despite the overall declines in CVD mortality rates over the past several decades, Black women aged 35 to 54 years are experiencing such declines at a much slower rate than women in other racial groups.^9^ Similar to what is described for Black women with breast cancer, several risk factors contribute to the disproportionate number of deaths associated with CVD within the Black community, including high rates of obesity, limited access to primary care clinicians, and other socioeconomic contributors.^10^

Radiation therapy (RT) is a vital part of adjuvant treatment in the management of breast cancer, particularly in women with locally advanced or recurrent disease, when the clinical target volume can include the breast and chest wall as well as surrounding nodal areas. Despite the locoregional and distant disease-free survival benefit of RT in patients with advanced breast cancer, there is a valid concern about toxicities to surrounding organs at risk; in particular, cardiovascular toxicity, primarily due to the proximity of the left anterior descending coronary artery (LAD) to the chest wall in cases of left-sided breast irradiation.^11-14^ Rates of major coronary events have been shown to increase linearly, with an average of 7.4% increase per Gray of radiation dose to the heart, in addition to a 16.3% increase in CVD within the first 4 years and a 15.5% increase after 5 to 9 years in patients with breast cancer compared with patients who did not receive RT. Furthermore, these cardiovascular events occurred with greater frequency in women with underlying cardiac risk factors than in those women without such factors.^15^

Traditional radiation techniques for locally advanced breast cancer include 3-dimensional conformal RT, which can frequently result in time-consuming, complex plans that can still lead to high doses of radiation to the heart and lungs, often having to sacrifice target coverage to protect these organs at risk.^16^ Newer technologies, including intensity-modulated RT or volumetric modulated arc therapy, have shown superior, homogeneous target coverage but frequently at the expense of a higher integral dose of radiation to cardiac structures.^17^^,^^18^ Pencil-beam scanning is the newest form of proton therapy and has been shown to be dosimetrically superior to photon therapy in the treatment of patients with breast cancer, offering excellent target coverage as well as a lower integral dose of radiation to critical surrounding structures, such as the heart, LAD, left ventricle, and lungs.^19-21^ Mitigating radiation dose to cardiac substructures with proton therapy can reduce the predicted risk of cardiac toxicity by up to 2.9% compared with conventional techniques, and this risk attenuation is most likely amplified in women who present with co-morbid conditions that already place them at risk for future cardiovascular events.^21^^,^^22^

Because Black women diagnosed with breast cancer tend to present with more advanced and aggressive disease, they also have a higher likelihood of requiring additional systemic cardiotoxic treatment modalities, such as anthracycline-based chemotherapies.^23^^,^^24^ The addition of systemic chemotherapy for breast cancer, along with an inherent increased risk of baseline CVD, makes it absolutely critical for Black women to have access to cardiac-sparing treatment modalities to mitigate their risk for cardiovascular events after cancer treatment is complete. There is a paucity of data in the literature investigating the cardiovascular profile of women undergoing proton therapy at a center that serves a predominantly Black, urban community.^25^ Furthermore, the Radiotherapy Comparative Effectiveness (RadComp) study, a phase 3 randomized trial that has recently closed to accrual, is investigating the potential benefit of proton therapy vs photon RT in decreasing cardiovascular events in women who require comprehensive nodal irradiation for breast cancer treatment^26^; however, it remains unclear whether there is a fair representation of Black women enrolled, and it can take years before initial findings will be discussed.^27^^,^^28^ Therefore, in this study, we investigated the baseline cardiovascular risk in women receiving proton therapy for breast cancer and compare cardiac and pulmonary events after RT is complete to determine the potential benefit of proton therapy for Black women compared with women in other racial groups. In our institution, all patients with breast cancer are equally considered for proton therapy, particularly if they have received prior RT, have left-sided locally advanced disease requiring comprehensive nodal irradiation, or present with substantial baseline CVD or anatomic variations that exceed radiation constraints to organs at risk using photon techniques.

## Methods

### Patient selection and study design

A retrospective analysis of 599 patients consecutively treated for breast cancer who underwent proton therapy at the Maryland Proton Treatment Center (MPTC) from June 2016 to December 2021 was conducted under institutional review board approval (HP-00080638). Patients were included in the analysis if they were 18 years of age or older with a pathologically proven invasive mammary carcinoma (ductal, lobular, or other) of the breast and received breast/chest wall with or without nodal (axillary, supraclavicular, or internal mammary nodes) irradiation at our center. Patients who had received prior RT or had disease recurrence were also included. Documentation of a cardiopulmonary condition was defined as the presence of 1 or more of the following: stroke, myocardial infarction, arrhythmias, cardiomyopathies, congestive heart failure, aortic aneurysm, pacemaker or implantable cardioverter-defibrillator placement, coronary artery disease, cardiac valve disease, pericardial effusion, asthma, chronic obstructive pulmonary disease, pulmonary embolism, acute respiratory distress syndrome, acute hypoxic respiratory failure, bronchiolitis obliterans organizing pneumonia, malignant pleural effusion, radiation-induced pulmonary fibrosis, or radiation pneumonitis. All patients were seen by a multidisciplinary oncology team that included breast surgeons, medical oncologists, and radiation oncologists. All proton therapy plans were designed using RayStation and dose-volume histogram curves were also extracted for each patient using this treatment planning system.

### Statistical analysis

We retrospectively collected patient demographics, socioeconomic characteristics, medical history, and cancer treatment from our electronic health records. We studied the association between patient cohorts (Black vs non-Black) and clinical parameters using Χ^2^ testing for categorical variables. The Mann-Whitney *U* test was used to assess differences between continuous variables. Overall survival was calculated from the date of first visit to MPTC to time of death or date of last follow-up. Freedom from recurrence was also determined from the date of first visit to MPTC to the time of first failure (local, regional, or distant), identified by imaging, biopsy, or clinical exam. The Kaplan-Meier method with log-rank testing was used to estimate overall survival and freedom from recurrence in the Black and non-Black patient groups. Binary logistic regression with forward modeling selection was used for multivariate analysis to identify factors associated with new cardiac and pulmonary events or conditions after proton therapy. Cox regression with forward modeling was used for multivariate analysis to identify factors associated with overall survival.

Variables analyzed in the Cox and binary logistic regression analysis include age (continuous), race (Black vs non-Black), sex, marital status (single vs not single), median income (continuous) estimated from the patient’s home zip code, insurance (yes vs no), ECOG-ACRIN performance status (continuous), diagnosis of type 2 diabetes (yes vs no), hypertension (yes vs no) or hyperlipidemia (yes vs no) at time of consultation and latest follow-up, current smoker (yes vs no), prior smoker (yes vs no), body mass index at consultation and latest follow-up (continuous), additional cardiac or cardiopulmonary co-morbidities at time of consultation (yes vs no), development of new cardiac or pulmonary conditions after proton therapy, histology (ductal carcinoma in situ vs invasive ductal carcinoma vs invasive lobular carcinoma vs other), estrogen receptor status (positive vs negative), ERBB2 (formerly HER2; positive vs negative), triple-negative disease (yes vs no), cancer stage, laterality (right vs left vs bilateral vs other), recurrent disease (yes vs no), prior RT to the contralateral or ipsilateral breast, neoadjuvant chemotherapy received (yes vs no), adjuvant chemotherapy given (yes vs no), concurrent chemotherapy given (yes vs no), total RT dose received (Gy), endocrine therapy received (yes vs no), total length of endocrine therapy given at date of last follow-up (continuous), calcification of the LAD seen (yes vs no), and dosimetric parameters summarized in Table S1 as continuous variables. All statistical analyses were performed using SPSS Statistics, version 29 (IBM Corp), and tests of statistical significance were 2 sided, with P less than .05 being statistically significant.

## Results

### Patient characteristics

A total of 599 patients were included in the study cohort; patient sociodemographic, disease, and treatment characteristics between the 2 cohorts are summarized in Table 1. Overall, 189 (32%) of patients self-identified as Black, and the majority (337/599 [56%]) of the non-Black cohort were predominantly White. Median follow-up for the entire cohort was 26 months (range = 0.47-90 months): 25 months (range = 0.8-82 months) for Black patients and 28 months (range = 0.47-90 months) for non-Black patients. In this study, Black patients were more likely to be single (41% vs 18%; *P* < .001) and reside in zip codes with a lower median income (*P* < .001). The Black cohort also had higher rates of hypertension (*P* < .001), additional cardiopulmonary conditions (*P* < .001), and higher median body mass index (*P* = .015) at the time of initial proton consultation, and this trend continued to the time of the latest follow-up after proton therapy was given (Table 1).

**Table 1.** Patient sociodemographic, cancer, and treatment characteristics (N = 599)

Characteristic	Black patients	Non-Black patients	*P*
	(n = 189)	**(n = 410)** ^a^	
Age, median (range), y	57 (19-88)	57 (28-91)	.758
Sex, No. (%)			.508
Female	187 (98.9)	403 (99.3)	
Male	3 (1.1)	3 (0.7)	
Marital status,^b^ No. (%)			<.001
Single	76 (40.6)	71 (17.8)	
Married	77 (41.2)	266 (66.8)	
Divorced	21 (11.2)	40 (10.1)	
Widowed	13 (7)	21 (5.3)	
Insurance,^c^ No. (%)			.593
Private	129 (68.3)	283 (70.2)	
Medicare/Medicaid	51 (27)	103 (25.6)	
Other	7 (3.7)	8 (2)	
Combination	2 (1)	8 (2.0)	
None	0 (0)	1 (0.2)	
Income, median (range)	$68 942 ($28 549-$155 000)	$94 135 ($29 196-$986 840)	<.001
ECOG-ACRIN performance status, median (range)	0 (0-3)	0 (0-3)	.070
Comorbidities at initial visit,^d^ No. (%)			
Hypertension	91 (48.1)	132 (32.5)	<.001
Diabetes	29 (15.3)	48 (11.8)	.240
Hyperlipidemia	46 (24.3)	96 (23.6)	.465
Additional cardiopulmonary conditions^e^	47 (24.9)	51 (12.6)	<.001
Body mass index at consultation, median (range), kg/m^2^	29.73 (18.85-49.53)	28.14 (16.52-5.98)	.015
Smoking status, No. (%)			
Current	12 (6.4)	19 (4.7)	.429
Former	43 (22.9)	103 (25.4)	.540
Type of surgery (before proton therapy),^f^ No. (%)			.058
None	10 (5.4)	7 (1.8)	
Partial mastectomy	67 (36.2)	156 (39.1)	
Mastectomy	96 (51.9)	194 (48.6)	
Local excision	8 (4.3)	33 (8.3)	
Other	4 (2.2)	9 (2.2)	
Histology,^g^ No. (%)			.698
Ductal carcinoma in situ	3 (1.6)	8 (2)	
Invasive ductal carcinoma	161 (85.2)	345 (85.2)	
Invasive lobular carcinoma	14 (7.4)	36 (8.8)	
Other	11 (5.8)	16 (4)	
American Joint Committee on Cancer, 8th ed, staging,^h^ No. (%)			.113
0	4 (2.1)	8 (2)	
I	22 (11.8)	59 (14.6)	
II	49 (26.2)	145 (35.8)	
III	54 (28.9)	95 (23.5)	
IV	12 (6.4)	15 (3.7)	
Recurrence	46 (24.5)	83 (20.5)	
Prior RT to treated area, No. (%)	45 (23.8)	66 (16.3)	.032
Prior RT to contralateral area, No. (%)	13 (6.9)	15 (3.7)	.098
Laterality,^g^ No. (%)			.255
Right	55 (29.3)	102 (25.1)	
Left	113 (60.1)	269 (66.3)	
Bilateral	19 (10.1)	35 (8.6)	
Nodal/mediastinum area	1 (0.5)	0 (0)	
Estrogen receptor positive, No. (%)	118 (62.8)	305 (75.5)	.002
Triple-negative disease, No. (%)	62 (33)	67 (16.6)	<.001
ERBB2 positive, No. (%)	39 (20.9)	93 (23.3)	.596
Neoadjuvant chemotherapy given, No. (%)	98 (51.9)	177 (43.7)	.039
Concurrent chemotherapy given, No. (%)	21 (11.2)	57 (14.1)	.362
Adjuvant chemotherapy given, No. (%)	54 (28.7)	133 (32.9)	.343
Total proton therapy dose, median (range), Gy	59.4 (3.0-72.0)	52.6 (3.6-78.4)	.406
Co-morbidities at follow-up, No. (%)			
Hypertension	114 (60.3)	136 (33.6)	<.001
Diabetes	42 (22.2)	35 (8.7)	<.001
Hyperlipidemia	53 (28)	92 (22.8)	.183
New cardiac events/conditions, No. (%)	15 (7.9)	23 (5.7)	.190
New pulmonary events/conditions, No. (%)	21 (11.1)	24 (5.9)	<.001
Body mass index at follow-up, median (range), kg/m^2^	31.37 (19.09-50.7)	27.2 (17.44-49.06)	<.001
Endocrine therapy use, No. (%)	104 (55)	289 (72.8)	<.001
Length of endocrine therapy use, median (range), mo	24 (1-120)	26 (1-240)	.303

Abbreviation: RT = radiation therapy.

a Non-Black patients include White (n = 337 [56.3%]), Other = Asian-Pacific patients, Latine and other ethnic groups (n = 69 [11.5%]), and Unknown (n = 4 [0.7%]).

b Missing: n = 14 patients.

c Missing: n = 7 patients.

d Co-morbidities seen at initial consultation for proton therapy.

e Cardiac and pulmonary conditions/events include history of stroke, myocardial infarction, arrhythmias, cardiomyopathies, congestive heart failure, aortic aneurysm, pacemaker/implantable cardioverter-defibrillator placement, coronary artery disease, cardiac valve disease, pericardial effusion, asthma, chronic obstructive pulmonary disease, pulmonary embolism, acute respiratory distress syndrome, acute hypoxic respiratory failure, bronchiolitis obliterans organizing pneumonia, malignant pleural effusion, radiation-induced pulmonary fibrosis, and radiation pneumonitis.

f Missing: n = 15 patients.

g Missing: n = 5 patients.

h Missing: n = 7 patients.

Regarding disease and treatment characteristics, Black women were more likely to present with triple-negative disease (*P* < .001) and receive neoadjuvant chemotherapy (*P* = .039) in addition to having received prior RT to the treated area receiving proton treatment (*P* = .032). On the contrary, the non-Black (predominantly White) patients had higher rates of estrogen receptor–positive disease (*P* = .002) and were subsequently more likely to use endocrine therapy (*P* < .001); yet, there was no difference in the total length of time of endocrine therapy use between the 2 groups (Table 1). There was no difference in terms of histology, disease stage, cancer laterality, and total proton therapy dose delivered between the 2 cohorts. After proton treatment, Black women were significantly more likely to develop a new pulmonary condition (*P* = .001).

We analyzed all the aforementioned patient social and demographic history, cancer, and treatment characteristics, including dose-volume histogram data (Table S1), to determine important predictors for cardiac and pulmonary events in our cohort. In the final multivariate model, patients diagnosed with hypertension and type 2 diabetes at the time of their most recent follow-up were approximately 4 and 2.6 times more likely, respectively, to have a cardiac event or condition after proton therapy was complete (Table 2). Patients were 2.6 times more likely to have a pulmonary condition or event after proton therapy if they self-identified as Black (Table 2). Recurrent disease was also associated with a nearly 5-fold increased risk and performance status, with a nearly 2-fold increased risk of a patient experiencing a pulmonary event after proton therapy.

**Table 2.** Factors associated with new cardiac and pulmonary events/conditions after proton therapy in the final multivariable model^a^

Characteristic	Odds ratio (95% CI)	*P*
Cardiac event/condition^b^		
Hypertension at follow-up	4.21 (1.93 to 9.18)	<.001
Diabetes at consultation	2.55 (1.17 to 5.53)	.018
Pulmonary event/condition^c^		
Black race^d^	2.60 (1.39 to 4.88)	.003
ECOG-ACRIN performance status	1.86 (1.21 to 2.86)	.004
Recurrent disease	4.98 (2.66 to 9.35)	<.001

a Binary logistic regression with forward model selection was used for the multivariate analysis.

b Cardiac events include stroke, myocardial infarction, arrhythmias, cardiomyopathies, congestive heart failure, aortic aneurysm, pacemaker/implantable cardioverter-defibrillator placement, coronary artery disease, cardiac valve disease, and pericardial effusion.

c Pulmonary events include asthma, chronic obstructive pulmonary disease, pulmonary embolism, acute respiratory distress syndrome, acute hypoxic respiratory failure, bronchiolitis obliterans organizing pneumonia, malignant pleural effusion, radiation-induced pulmonary fibrosis, and radiation pneumonitis.

d Compared with patients of other racial groups.

### Dose-volume histogram characteristics

Dosimetric parameters derived from dose-volume histogram curves were compared between the Black and non-Black patient cohorts (Table S1). Several heart dosimetric parameters were higher in Black women than in the other groups, including mean (*P* = .038; average dose <1 Gy for both cohorts) and maximum dose delivered to the heart (*P* = .021), as well as the volume of the heart receiving 10 Gy (*P* = .037), 30 Gy (*P* = .026), 40 Gy (*P* = .005), and 50 Gy (*P* < .001). The mean total dose (*P* = .001) and maximum total dose (*P* = .028) delivered to the ipsilateral lung, however, as well as the volume of the ipsilateral lung that received 5 Gy (*P* = .028) and 20 Gy (*P* = .001), respectively, were all higher in the non-Black cohort. There were no significant differences in the dosimetric parameters of the LAD, left ventricle, and total lung between the 2 groups (Table S1).

### Overall survival

**Figure 1.**
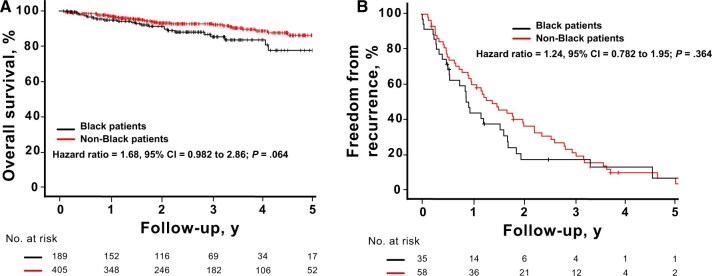
Comparison of overall survival and freedom from recurrence between Black patients and patients from other racial groups

Mean overall survival for the entire cohort was 83 months (median time could not be calculated; 95% CI: 80–86 months) with a 5-year overall survival rate of approximately 84%. There was no difference in overall survival between Black patients (mean overall survival = 71 months; 95% CI: 66–75 months); 5-year overall survival = 85%) and non-Black patients (mean overall survival = 84 months; 95% CI: 81–88 months); 5-year overall survival = 86%) (Figure 1, A). Statistically significant predictors of overall survival in the final multivariate analysis are summarized in Table 3. Patients were nearly 2 and 1.7 times more likely to die if they had a poorer ECOG-ACRIN performance status or higher cancer stage, respectively. The addition of new cardiac or pulmonary events after proton therapy increased the risk of death approximately 3-fold and 6-fold, respectively (Table 3), whereas length of receiving endocrine therapy was associated with a relative 3% decreased risk of death. Interestingly, the volume of the LAD and the left ventricle receiving 5 and 23 Gy, respectively, were predictors of increased risk of death, at nearly 1.6-fold for the latter cardiac substructure (Table 3).

**Table 3.** Factors associated with overall survival in the final multivariable cox model^a^

Characteristic	Hazard ratio (95% CI)	*P*
ECOG-ACRIN performance status	2.15 (1.18 to 3.93)	.013
Cancer stage	1.68 (1.20 to 234)	.002
New cardiac events/conditions^b^	2.66 (1.06 to 6.65)	.037
New pulmonary events/conditions^c^	6.43 (2.58 to 16.03)	<.001
Length of endocrine use, mo	0.969 (0.951 to 0.988)	.013
Volume of the left anterior descending coronary artery receiving at least 5 Gy	1.001 (1.00 to 1.001)	.041
Volume of the left ventricle receiving at least 23 Gy	1.57 (1.27 to 1.94)	<.001

a Cox regression with forward model selection was used for the multivariate analysis.

b Cardiovascular events include stroke, myocardial infarction, arrhythmias, cardiomyopathies, congestive heart failure, aortic aneurysm, pacemaker/implantable cardioverter-defibrillator placement, coronary artery disease, cardiac valve disease, and pericardial effusion.

c Pulmonary events include asthma, chronic obstructive pulmonary disease, pulmonary embolism, acute respiratory distress syndrome, acute hypoxic respiratory failure, bronchiolitis obliterans organizing pneumonia, malignant pleural effusion, radiation-induced pulmonary fibrosis, and radiation pneumonitis.

### Freedom from recurrence and pattern of failure

There was no difference in freedom from recurrence between the 2 cohorts, where the median time to failure for Black and non-Black patients was 11 and 18 months, respectively (Figure 1, B) (*P* = .364). Patterns of failure between groups do differ (Table 4), however, where Black women were more likely to fail with distant or combination tumor recurrence compared with the other cohort. Locoregional failures were less than 5% for all patients.

**Table 4.** Patterns of failure (N = 595)^a^

Site of first failure	Black patients, No. (%)	Non-Black patients, No. (%)	*P*
	(n = 189)	(n = 406)	
No evidence of disease	144 (76.2)	339 (83.5)	.043
Locoregional	7 (3.7)	15 (3.7)	
Distant	32 (16.9)	49 (12.1)	
Combination	6 (3.2)	3 (0.7)	

a Missing: n = 4 patients.

## Discussion

To the best of our knowledge, this is the first study to intentionally center the experience of Black women undergoing proton therapy as part of their breast cancer care, comparing the cardiopulmonary and treatment profiles of this historically marginalized group with other groups that do not traditionally share similar health inequities. Our findings confirm that Black patients with breast cancer present with more aggressive disease at treatment presentation, requiring more cardiotoxic systemic therapy (Table 1), and subsequently are 2.6 times more likely to experience pulmonary toxicity, even after receiving pencil beam scanning proton therapy (Table 2). No identified dose-volume histogram parameter predicted cardiac toxicities in the cohort that received pencil beam scanning proton therapy, but a diagnosis of diabetes before RT and the development of hypertension at follow-up increased the risk of cardiac events 2.5- to 4-fold in this cohort (Table 2). Nearly half of our Black patients presented at our MPTC diagnosed with hypertension, and this rate increased by 10% after proton therapy was completed, alongside a 7% absolute increase in diabetes diagnoses after RT (Table 1). Patients with cancer who received chemotherapy and radiation can often experience accelerated ageing and are at high risk of developing co-morbidities that can lead to cardiovascular events, negatively affecting outcomes.^29-32^ Indeed, cardiac events in our analysis increased the risk of death nearly 3-fold (Table 3), and Black women disproportionately experience such co-morbid conditions. This finding highlights the critical role of communicating healthy lifestyle changes in this patient population, perhaps engaging survivorship programs that focus on sustainable nutrition goals and physical exercise.^33^

In our analysis, race was not predictive of cancer survival outcomes (Figure 1, A and B), but the volume of the LAD receiving at least 5 Gy and the volume of the left ventricle receiving at least 23 Gy were associated with increased mortality risk after breast cancer radiation with pencil beam scanning proton therapy. Limited data are currently available regarding the relationship between cardiac substructure radiation exposure and the development of short-term and long-term clinical toxicities and survival outcomes for patients, especially when using proton therapy.^34^^,^^35^ Advances in imaging and contouring platforms have made it increasingly feasible to delineate the substructures of the heart when creating radiation plans for patients with breast cancer.^36-38^ These advanced plans have demonstrated that dose distribution to the heart is not homogenous, with higher doses often delivered to the apex and apical anterior segments.^39^ A Swedish study further confirmed these differences, revealing a statistically significant increase in LAD stenosis in patients with left-sided breast cancer who received RT compared with patients with right-sided breast cancer.^14^ Thus, mean heart dose alone may be an inadequate dosimetric parameter for assessing RT-associated cardiotoxicity and dose-response relationships. The BreAst Cancer and CArdiotoxicity Induced by RAdioTherapy (BACCARAT) study revealed that more than 55% of patients with left-sided breast cancer receiving mean heart dose less than 3 Gy could still be exposed to LAD doses greater than 40 Gy. Furthermore, the left ventricle and LAD were the most exposed substructures of the heart, with mean doses of 6.2 Gy and 15.7 Gy, respectively.^40^ From a clinical standpoint, left ventricular systolic function plays a vital role in defining the evaluation and management of cardiac disease and in determining patient prognosis.^41^ Similar to the predictive value of left ventricular dose in our study, van den Bogaard et al.^42^ concluded that the dose to the left ventricle, particularly a dose of at least 5 Gy, was a better predictor of acute coronary events than mean heart dose or dose to other heart substructures. Associations between LAD dose and increased risk of cardiac events and all-cause mortality in patients with non–small cell lung cancer have also been well established.^43^ Interestingly, there were differences in the dosimetry seen in certain cardiac substructures between the 2 racial cohorts (Table S1). Our physicians and dosimetry team use standard approaches to contour cardiac substructures, so the difference calculated is most likely attributed to anatomical variations.^44^ Generous treatment of internal mammary nodes in Black women due to more aggressive disease and varying use of inspiratory breath-holding techniques, however, may also have contributed to the differences.^45^

Our study findings provide more evidence of the importance of minimizing dose distribution to individual cardiac substructures, supporting the use of advanced radiation techniques such as deep inspiratory breath holding with photon treatment strategies or proton therapy to spare important organs at risk, such as the heart and lungs, especially in high-risk patients who have existing cardiovascular risk factors.^46^ An increase in the accessibility and implementation of advanced radiation technology in this high-risk subset of patients with breast cancer has the potential to decrease the overall burden of cardiopulmonary toxicity and improve clinical outcomes. Across the country, proton therapy remains a scarce and high-cost resource, especially for minority and marginalized populations residing in low socioeconomic areas.^25^ A cross-sectional analysis led by the American Cancer Society found that across the 45 proton facilities across the country, Black patients were 33% less likely to be treated with proton therapy than White patients, especially for cancers for which proton therapy is recommended over photon-based RT (odds ratio = 0.67, 95% CI = 0.64 to 0.71). They also found that these disparities increased over time, despite increases in the number of facilities offering proton therapy in the United States (annual percentage change = 0.09, *P* < .001).^25^

Beyond accessibility to proton therapy, Black women are more likely than women in other racial groups to experience treatment delays and discontinuation as well as longer intervals between abnormal screening mammogram findings and follow-up; they are also less likely to receive guideline-concordant care, even after controlling for insurance status.^47^^,^^48^ To provide equitable services for Black patients, radiation oncologists and hospital systems must prioritize efforts to mitigate these access barriers. Kronfli et al.^49^ demonstrated that Black patients undergoing RT were more than 3 times more likely than their counterparts in other racial groups to have unmet transportation needs (64% vs 19%; *P* < .001). Hypofractionated therapy—the delivery of large doses of radiation over fewer courses of treatment—can serve as a potential solution by increasing convenience and minimizing transportation burdens for patients, improving continuity of care and follow-up. Several studies have validated the safety and efficacy in clinical outcomes of various hypofractionated treatment models compared with standard fractionated regimens.^50^^,^^51^ In addition, the coordination of medical appointments among various cancer team members through same-day visits can help minimize transportation needs. The establishment of cost-neutral payment models to match the cost of proton therapy with that of conventional RT can improve affordability for vulnerable populations who present with increased financial barriers and assistance needs.^49^^,^^52^ Such collaborative payor environments promote equitable access to advanced radiation treatment modalities for all patients.

This study does have some limitations. First, it was conducted at a single academic institution with a short follow-up period of 26 months. We did not find any significant differences in the development of new cardiac events, overall survival, or freedom from recurrence between the 2 groups; however, this may be attributed to the longer natural history of CVD development and the current lack of sufficient follow-up data. Further study is needed to explore the longer-term development of new cardiac and pulmonary events as well as survival outcomes. Second, nearly one-quarter of our patients underwent previous radiation for a breast malignancy. For the majority of those patients, we did not have access to their previous radiation records and were unable to calculate a total cumulative dose of radiation on important organs at risk, possibly confounding our results. Third, a limited number of patients with stage IV disease were also included in our analysis, and though not considered curable, due to advances in systemic treatment options, patients with limited stage IV breast cancer are living longer and may develop cardiac or pulmonary events, as we tried to characterize.^53^^,^^54^ Finally, because of the limitations of a retrospective chart review, we were unable to discern which patients underwent proton therapy due to exceeding organs-at-risk constraints, missing an opportunity to characterize in which dosimetric scenarios proton therapy may be more beneficial in our 2 racial subgroups.

Nevertheless, our study demonstrates that Black patients with breast cancer endure a higher incidence of cardiopulmonary events and high-risk co-morbidities both before and after RT; they also present with more unfavorable disease characteristics. As a result, we theorize that Black patients with breast cancer and a baseline increased CVD risk may derive greater therapeutic benefit from the organ-sparing effects of proton therapy than patients in other racial groups. RadComp will hopefully shed light as to the benefit of proton therapy and its effects on mitigating cardiovascular events, especially within the Black community. Future efforts focused on improving the accessibility of care for Black and other minority populations are imperative to mitigate the ongoing racial inequities in breast cancer outcomes.

## Supplementary Material

pkae129_Supplementary_Data

## Data Availability

Research data are stored in an institutional repository and will be shared upon request to the corresponding author.
