# Supplementary material for: Cancer outcomes and cardiopulmonary toxicities for Black patients with breast cancer treated with proton therapy
Source: JNCI Cancer Spectr. 2024 Dec 28;9(1):pkae129. doi: 10.1093/jncics/pkae129 (PMC12257934; doi:10.1093/jncics/pkae129)
Supplement: pkae129_Supplementary_Data [file pkae129_supplementary_data.zip › Supplemental Materials.docx]

| **Supplemental Table 1.** Dose volume histogram (DVH) dosimetric parameters | | | |
| --- | --- | --- | --- |
| **Dosimetric Parameter** | **Black patients**  **(n=150)^a^**  Mean (range) | **Non-Black patients**  **(n=334)^b^**  Mean (range) | **p-Value** |
| **Heart** |  |  |  |
| Mean total dose (Gy) | 0.992 (0-0.834) | 0.866 (0-0.672) | **0.038** |
| Max total dose (Gy) | 38.91 (0-70.20) | 37.50 (0-67.90) | **0.021** |
| V5^c^ (%) | 4.67 (0-29.59) | 4.26 (0-19.62) | 0.056 |
| V10 (%) | 2.88 (0-23.54) | 2.43 (0-13.28) | **0.037** |
| V20 (%) | 1.38 (0-16.94) | 1.06 (0-8.44) | 0.052 |
| V30 (%) | 0.739 (0-12.08) | 0.498 (0-6.63) | **0.026** |
| V40 (%) | 0.375 (0-7.70) | 0.205 (0-5.17) | **0.005** |
| V50 (%) | 0.100 (0-2.88) | 0.041 (0-3.87) | **<0.001** |
| **Left anterior descending artery (LAD)** | | | |
| Mean total dose (Gy) | 4.28 (0-31.85) | 3.33 (0-24.91) | 0.281 |
| Max total dose (Gy) | 16.26 (0-69.62) | 14.91 (0-50.61) | 0.788 |
| V5 (%) | 22.32 (0-81.93) | 32.35 (0-4610) | 0.164 |
| V15 (%) | 9.86 (0-67.25) | 7.66 (0-56.60) | 0.187 |
| V30 (%) | 2.86 (0-58.5) | 1.62 (0-50.4) | 0.161 |
| V40 (%) | 1.29 (0-54.16) | 0.419 (0-40.49) | 0.212 |
| **Left Ventricle (LV)** | | | |
| Mean LV dose (Gy) | 0.355 (0-6.98) | 0.275 (0-3.18) | 0.427 |
| Max LV dose (Gy) | 14.19 (0-69.55) | 12.72 (0-53.21) | 0.802 |
| V5 (%) | 1.60 (0-28.71) | 1.24 (0-17.65) | 0.372 |
| V15 (%) | 0.463 (0-16.71) | 0.271 (0-6.58) | 0.132 |
| V23 (%) | 0.214 (0-11.85) | 0.094 (0-3.14) | **0.011** |
| **Ipsilateral lung** | | | |
| Mean total dose (Gy) | 6.88 (0-20.55) | 7.61 (0.02-15.73) | **0.001** |
| Max total dose (Gy) | 47.79 (0-69.30) | 49.22 (1.50-74.58) | **0.028** |
| V5 (%) | 34.42 (0-75) | 37.19 (0-63.75) | **0.028** |
| V20 (%) | 12.25 (0-43.5) | 13.93 (0-35.63) | **0.001** |
| **Total lung** | | | |
| Mean total dose (Gy) | 4.03 (0.01-10.37) | 4.03 (0-11.55) | 0.925 |
| Max total dose (Gy) | 49.48 (0.94-69.92) | 48.20 (0.05-74.58) | 0.951 |
| V5 (%) | 19.90 (0-48.50) | 20.22 (0-163) | 0.910 |
| V20 (%) | 7.08 (0-21.10) | 7.096 (0-23.50) | 0.969 |
| Abbreviations: Gray (Gy)  *^a^*Missing: n=72 patients;  *^b^*Missing: n=39 patients;  *^c^*VX = Volume (%) of corresponding organ receiving X amount of gray (Gy) | | | |

**Supplementary materials**
